# Drimane Sesquiterpene-Conjugated Amino Acids from a Marine Isolate of the Fungus *Talaromyces minioluteus* (*Penicillium Minioluteum*)

**DOI:** 10.3390/md13063567

**Published:** 2015-06-05

**Authors:** Suthatip Ngokpol, Wittaya Suwakulsiri, Sanya Sureram, Kriengsak Lirdprapamongkol, Thammarat Aree, Suthep Wiyakrutta, Chulabhorn Mahidol, Somsak Ruchirawat, Prasat Kittakoop

**Affiliations:** 1 Chulabhorn Research Institute, Kamphaeng Phet 6 Road, Laksi, Bangkok 10210, Thailand; E-Mails: ngokpol@hotmail.com (S.N.); sanya@cri.or.th (S.S.); kriengsak@cri.or.th (K.L.); mahidol_natlab@cri.or.th (C.M.); somsak@cri.or.th (S.R.); 2 Chulabhorn Graduate Institute, Chemical Biology Program, Laksi, Bangkok 10210, Thailand; E-Mail: 13020202@cgi.ac.th; 3 Department of Chemistry, Faculty of Science, Chulalongkorn University, Bangkok 10330, Thailand; E-Mail: thammarat.aree@gmail.com; 4 Department of Microbiology, Faculty of Science, Mahidol University, Bangkok 10400, Thailand; E-Mail: suthep.wiy@mahidol.ac.th; 5 Center of Excellence on Environmental Health and Toxicology (EHT), CHE, Ministry of Education, Bangkok 10400, Thailand

**Keywords:** marine fungi, *Talaromyces minioluteus*, *Penicillium minioluteum*, sesquiterpene, drimane, caspase-3, cytotoxic activity, fungal metabolites

## Abstract

Four new sesquiterpene lactones (**3**, **4**, **6** and **7**) and three known compounds, purpuride (**1**), berkedrimane B (**2**) and purpuride B (**5**), were isolated from the marine fungus, *Talaromyces minioluteus* (*Penicillium minioluteum*). New compounds were drimane sesquiterpenes conjugated with *N*-acetyl-l-valine, and their structures were elucidated by analysis of spectroscopic data, as well as by single crystal X-ray analysis. The isolated compounds could not inhibit the apoptosis-regulating enzyme, caspase-3, while three of the compounds (**2**, **3** and **7**) exhibited weak cytotoxic activity.

## 1. Introduction

**Figure 1:**
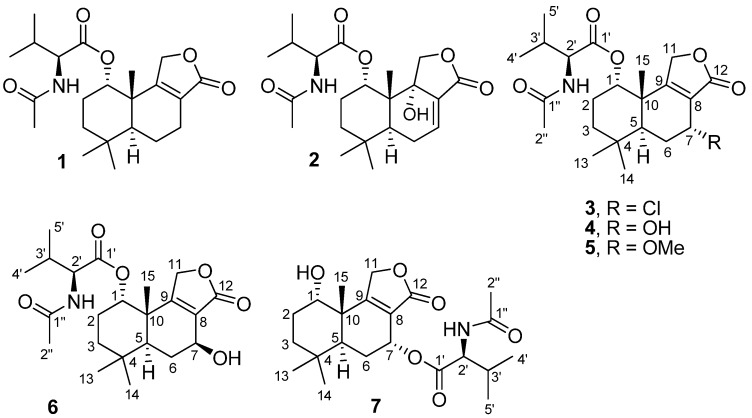
Structure of fungal metabolites **1**–**7**.

Marine fungi produce a diverse array of secondary metabolites that exhibit various biological activities [1,2], and they are rich sources of antitumor agents [3]. Fungi of the genus *Penicillium* are rich in bioactive compounds [4,5,6,7,8], and among them, *Penicillium minioluteum* produces bioactive metabolites, for example, polyketide-terpenoid hybrids [9], hydrazide derivative as a potentiator of nerve growth factor [10], and antifungal tetracyclic diterpenes [11]. The fungus *P. minioluteum* was recently found to produce isomeric furanones through epigenetic manipulation through the addition of azacitidine, a DNA methyltransferase inhibitor [12]; it was also used in the biotransformation of clovane derivatives for the synthesis of rumphellclovane A [13]. It should be noted that the name of the fungus *P. minioluteum* was recently revised to “*Talaromyces minioluteus*” [14]. Constituents in the genus *Talaromyces* have been previously studied, such as nematocidal PKS-NRPS hybrid metabolites [15], nardosinane-type sesquiterpene [16], cytotoxic norsesquiterpene peroxides [17], and acetylcholinesterase inhibitors [18]. The present work reports the isolation and characterization of drimane sesquiterpene lactones (**1**–**7**) from the marine fungus *T. minioluteus* strain PILE 14-5 (Figure 1). Four new compounds (**3**, **4**, **6** and **7**) were sesquiterpene lactones conjugated with *N*-acetyl-l-valine, and such sesquiterpenes are rare in nature. So far, only five derivatives of a drimane conjugated with amino acids have been reported to date [19,20,21]. Cytotoxic activity and the inhibitory activity toward caspase-3 enzyme of **1**–**7** are also reported. 

## 2. Results and Discussion

### 2.1. Structure Elucidation of New Fungal Metabolites **3**, **4**, **6** and **7**

Separation of culture extracts of *T. minioluteus* by Sephadex LH-20 column chromatography and C_18_ reversed-phase HPLC yielded drimane sesquiterpene lactones including three know compounds, purpuride (**1**), berkedrimane B (**2**) and purpuride B (**5**), and four new metabolites, namely minioluteumides A–D (**3**, **4**, **6** and **7**) (Figure 1). Spectroscopic data of **1**, **2** and **5** were in good agreement with those reported in the literature [19,20,21].

Minioluteumide A (**3**) had a molecular formula, C_22_H_32_ClNO_5_, as deduced from the ESITOF MS. The MS isotopic pattern (the ratio of (M + H):(M + H + 2) = 3:1) revealed the presence of one chlorine atom in **3**. ^1^H NMR spectrum of **3** notably showed signals of six methyls (four singlets for H_3_-13, H_3_-14, H_3_-15 and H_3_-2′′ and two doublets for H_3_-4′ and H_3_-5′), three downfield methines (H-1, H-7, and H-2′), one methylene (H_2_-11) attached to an oxygen atom, an exchangeable N*H* proton, and a number of methines and methylenes at δ_H_ 1.41–2.40 (Table 1). ^13^C NMR spectrum of **3** showed 22 lines, and 

**Table 1:** ^1^H (600 MHz) and ^13^C (150 MHz) NMR data (CDCl_3_) for compounds **3** and **4**.

	3		4	
	δ_C_	δ_H_, multi (*J* in Hz)	δ_C_	δ_H_, multi (*J* in Hz)
1	74.3	5.02, brs	74.4	4.97, s
2	22.4	1.85, dq (2.9, 15.1); 1.95–2.07, m	22.4	1.83, dq (2.7, 15.1); 1.94, m
3	34.8	1.42, dt (3.8, 13.8); 1.65, td (4.0, 14.0)	34.9	1.38, dt (2.5, 13.7); 1.58, td (4.2,13.9)
4	32.2	-	32.3	-
5	39.8	2.40, d (12.4)	39.7	2.20, dd (1.1,12.4)
6	28.4	1.95–2.07, m; 2.22, d (14.8)	27.2	1.75, td (4.4, 13.9); 2.01, brd (14.1)
7	48.6	4.86, t (1.8)	59.6	4.55, brd (1.6)
8	126.4	-	127.1	-
9	170.8	-	170.8	-
10	41.5	-	41.4	-
11	67.8	4.60, d (17.6); 4.72, dd (1.9,17.6)	68.2	4.60, d (17.4); 4.72, dd (1.6,17.4)
12	170.8	-	173.4	-
13	32.7	1.06, s	33.0	1.04, s
14	21.8	0.97, s	21.4	0.96, s
15	20.2	1.24, s	19.6	1.20, s
1′	170.2	-	170.8	-
2′	58.2	4.42, dd (5.3, 8.2)	58.5	4.32, dd (6.0, 8.0)
3′	30.8	2.06, m	30.3	2.07, oct (6.5)
4′	18.0	0.89, d (6.6)	18.0	0.92, d (6.6)
5′	19.0	0.94, d (6.6)	19.1	0.95, d (6.6)
1″	170.3	-	170.5	-
2″	22.9	2.00, s	22.7	1.97, s
N*H*	-	5.74, d (8.0)	-	6.16, d (7.9)
OMe	-	-	-	-

**Figure 2:**
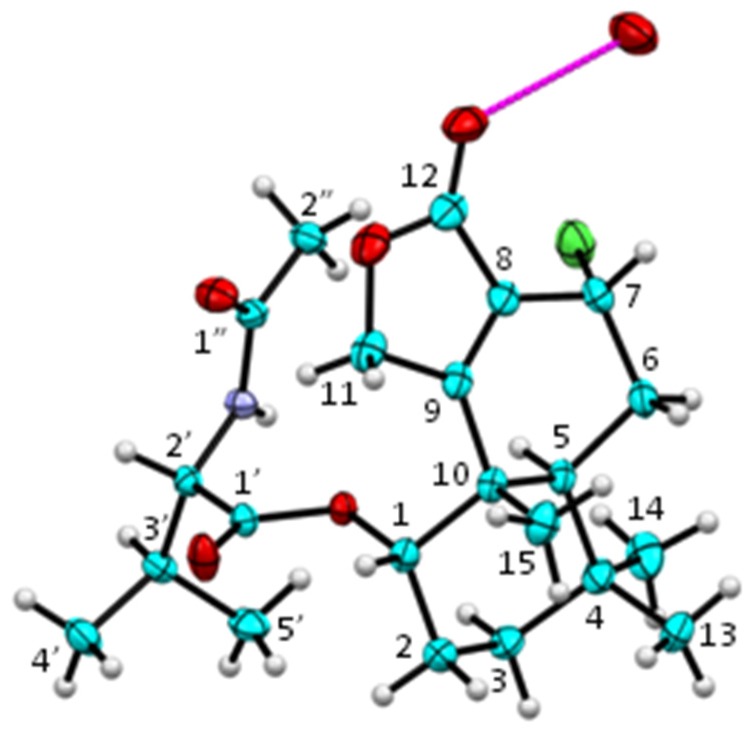
ORTEP plot (20% probability level) of **3** (color codes: C = cyan, O = red, N = purple, Cl = green, H = white). The molecule is stabilized by an intermolecular O–H···O hydrogen bond (magenta) with a disordered water molecule (occupancy factor 0.25).

DEPT spectra revealed the presence of six methyl, four methylene, five methine, and seven quaternary carbons. ^1^H–^1^H COSY spectrum established partial structures of H-1/H_2_-2/H_2_-3; H-5/H_2_-6/H-7; and H-2′/H-3′/H_3_-4′/H_3_-5′ in **3**. HMBC spectrum of **3** displayed correlations from H-1 to C-3 and C-10; H_2_-2 to C-4 and C-10; H_2_-3 to C-1, C-4, C-5, and C-14; H-5 to C-4, C-7, C-9, C-13, C-14, and C-15; H_2_-6 to C-4, C-8, and C-10; H-7 to C-5, C-8, C-9, and C-12; H_2_-11 to C-8, C-9, and C-12; both H_3_-13 and H_3_-14 to C-3, C-4, and C-5; and H_3_-15 to C-1, C-5, C-9, and C-10. These HMBC correlations, as well as the ^1^H–^1^H COSY correlations mentioned above, established a structure of a drimane sesquiterpene lactone, which was the same core structure as that of purpuride (**1**) [19]. The fragment of H-2′/H-3′/H_3_-4′/H_3_-5′ from the ^1^H–^1^H COSY spectrum, together with the HMBC correlations from H-2′ to C-1′; NH proton to C-1′, C-2′ and C-1′′; and H_3_-2′′ to C-1′′, established a structure of *N*-acetyl-valine in **3**. Chemical shifts (δ_H_ 5.02; δ_C_ 74.3) of H-1 in **3** suggested that C-1 was attached to an oxygen atom, and the downfield ^1^H resonance (δ_H_ 5.02) implied the presence of an ester (or ether) linkage at C-1. The HMBC correlation from H-1 to C-1′ readily indicated that the drimane unit was linked, through an ester bond at C-1, with the *N*-acetyl-valine residue. These spectroscopic data constructed a planar structure of **3**, and the assignment of ^1^H and ^13^C resonances in **3** is shown in Table 1. Small values of coupling constants for H-1 (brs) and H-7 (1.8 Hz) in **3** suggested that both H-1 and H-7 were equatorial. A single crystal of minioluteumide A (**3**) was subsequently subjected to an X-ray analysis, using CuKα radiation together with determining the Flack parameter [22]. It is known that an X-ray analysis using CuKα radiation with the Flack parameter provides a reliable determination of the absolute configuration [22]. Finally, the structure and absolute configuration of minioluteumide A (**3**) were confirmed by an X-ray analysis; the ORTEP plot is depicted in Figure 2. It should be noted that the amino acid in **3** is l-valine, which is also found in purpuride (**1**) and berkedrimane B (**2**) [19,20].

Minioluteumide B (**4**) exhibited a molecular formula of C_22_H_33_NO_6_ (by ESITOF MS), and its ^1^H and ^13^C NMR spectra shared a great deal of similarities with that of **3** (Table 1). A notable difference between ^1^H and ^13^C resonances at C-7 of **4** (δ_H_ 4.55; δ_C_ 59.6) and **3** (δ_H_ 4.86; δ_C_ 48.6) was observed. These data, as well as the molecular formula obtained from ESITOF MS, implied that a chlorine atom in **3** was replaced by a hydroxyl group in **4**. Partial structures of H-1/H_2_-2/H_2_-3; H-5/H_2_-6/H-7; and H-2′/H-3′/H_3_-4′/H_3_-5′ in **4** were obtained from ^1^H–^1^H COSY spectrum. Key HMBC correlations in **4** were observed from H-1 to C-3, C-5, C-10, and C-15; H_2_-3 to C-4, C-13, and C-14; H-5 to C-3, C-4, C-10, C-13, C-14, and C-15; H_2_-6 to C-4, C-8, and C-10; H-7 to C-8, C-9, and C-12; H_2_-11 to C-8, C-9, and C-12; both H_3_-13 and H_3_-14 to C-3, C-4, and C-5; and H_3_-15 to C-1, C-5, C-9, and C-10; these correlations secured the drimane core structure in **4**. Moreover, NMR signals of the *N*-acetyl-l-valine unit in **4** were almost identical to that of **3** (Table 1), and the HMBC correlation from H-1 to C-1′ established the ester linkage in **4**. The coupling constant of 1.6 Hz for H-7 in **4** was similar to that (1.8 Hz) for H-7 in **3**, suggesting that **4** had the same H-7 configuration as that of **3**. Minioluteumide B (**4**) should have the same biosynthetic origin as that of **3**, and therefore, they should share the same absolute configuration. Upon these spectroscopic data, the structure of **4** was established.

Minioluteumide C (**6**) had the same molecular formula, C_22_H_33_NO_6_, as that of **4**, and it also had similar ^1^H and ^13^C NMR spectra to that of **4**. Moreover, **6** showed similar ^1^H–^1^H COSY and HMBC correlations to that of **4**, suggesting that **6** had the same sesquiterpene lactone and *N*-acetyl-l-valine units as that in **4**. Although **6** had the same 7-OH group as that in **4**, detailed analysis of NMR data revealed a marked difference between the coupling constants of H-7 for **6** (t, 8.8 Hz) and **4** (brd, 1.6 Hz); these coupling constants indicated that H-7 in **6** and **4** had axial and equatorial orientations, respectively. Therefore, minioluteumide D (**6**) was a 7-epimer of **4**, and resonances of protons and carbons in **6** were assigned by analysis of 2D NMR spectra (Table 2).

Minioluteumide D (**7**) had a molecular formula, C_22_H_33_NO_6_, which was the same as that of **4** and **6**. However, ^1^H and ^13^C NMR spectra of **7** (Table 2) were different from those of **4** and **6**. Analysis of 2D NMR data revealed that **7** had the same sesquiterpene lactone and *N*-acetyl-l-valine units as that in **4** and **6**. ^1^H NMR resonance at H-1 of **7** was notably upfield shifted (δ_H_ 3.89) when compared to those of **4** (δ_H_ 4.97) and **6** (δ_H_ 5.00), while the H-7 signal in **7** was downfield (δ_H_ 5.71) as compared to those of **4** (δ_H_ 4.55) and **6** (δ_H_ 4.61); these data suggested that compound **7** had the *N*-acetyl-l-valine moiety attached at C-7, not at C-1 like that in **4** and **6**. The HMBC correlation from H-7 to C-1′ indicated the attachment of the *N*-acetyl-l-valine unit at C-7 of **7**. Small coupling constants of H-1 (brs) and H-7 (2 Hz) implied that both H-1 and H-7 in **7** were equatorial, similar to that in **4**. Upon these spectroscopic data, the structure of minioluteumide E (**7**) was established. Normally, the core structure of sesquiterpene lactones is attached to the *N*-acetyl-l-valine moiety at C-1 [19,20]; however, that of minioluteumide E (**7**) is connected to the amino acid at C-7, representing the new type of this compound class.

Although many drimane sesquiterpene lactones have been isolated to date [23,24], those conjugated with *N*-acetyl-l-valine are rare natural products; so far there have been only five natural derivatives, *i.e.*, purpuride (**1**), purpurides B and C, and berkedrimanes A and B. In 1973, purpuride (**1**) was first isolated from the fungus *Penicillium purpurogenum* [19], and forty years later, purpurides B and C were obtained from the same fungal species [21]. Berkedrimanes A and B (e.g., **2**) were isolated from the extremophilic fungus *Penicillium solitum* [20]. It is worth mentioning that the halogen-containing derivative, minioluteumide A (**3**), and the C-7 substituted amino acid, minioluteumide E (**7**), are the new representatives of this compound class.

**Table 2:** ^1^H (600 MHz) and ^13^C (150 MHz) NMR data (CDCl_3_) for compounds **6** and **7**.

	6		7	
	δ_C_	δ_H_, multi (J in Hz)	δ_C_	δ_H_, multi (J in Hz)
1	74.6	5.00, brs	71.0	3.89, brs
2	22.3	1.78, q (3.0); 1.96, m	25.7	1.63, dq (3.1,14.8); 2.06, m
3	34.5	1.36, dt (2.5, 15.2); 1.53-1.63, m	34.1	1.33, dt (3.7, 12.8); 1.74, td (4.1, 14.1)
4	32.6	-	32.5	-
5	44.4	1.81, brd (12.8)	39.1	2.06, brd (13.2)
6	27.1	1.53–1.63, m; 2.26, dd (6.9, 12.9)	25.6	1.86, ddd (15.0, 13.2, 4.5); 1.97, brd (15.0)
7	64.8	4.61, t (8.8)	62.9	5.71, t (2.0)
8	127.8	-	122.7	-
9	168.5	-	175.9	-
10	41.6	-	42.2	-
11	68.0	4.55, dd (2.7, 17.2); 4.73, d (17.2)	68.5	4.81, dd (2.0, 17.1); 5.11, d (17.1)
12	172.9	-	171.8	-
13	33.1	1.02, s	32.7	0.93, s
14	21.4	0.97, s	21.1	0.91, s
15	21.1	1.33, s	20.1	1.13, s
1′	171.2	-	170.8	-
2′	57.7	4.43, dd (4.8, 8.2)	57.3	4.56, dd (4.6, 8.7)
3′	30.6	1.96, m	31.2	2.17, otc (6.8)
4′	17.5	0.85, d (6.8)	17.6	0.90, d (6.6)
5′	19.0	0.92, d (6.8)	19.0	0.95, d (6.6)
1′′	170.0	-	169.7	-
2′′	23.0	2.01, s	23.1	2.02, s
N*H*	-	5.78, d (8.2)	-	6.04, d (8.6)

### 2.2. Biological Activities of the Isolated Compounds **1**–**7**

**Figure 3:**
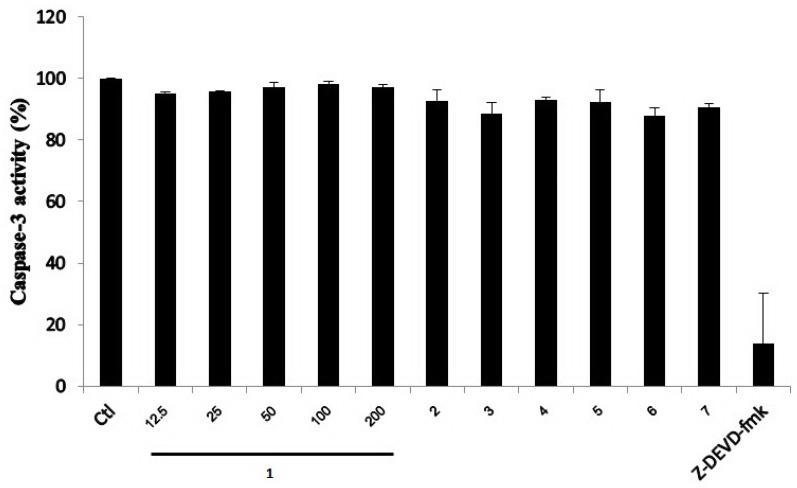
Effect of compounds **1**–**7** on caspase-3 enzymatic activity. Apoptotic HepG2 cell lysates were individually incubated with various concentrations (12.5–200 μM) of purpuride (**1**) and 200 µM of compounds **2**–**7**. A caspase-3 inhibitor (5 µM), Z-DEVD-fmk, was used as a positive control. Data are mean + SD of three independent experiments. Ctl = A control without compounds.

Previously, sesquiterpene lactones conjugated with *N*-acetyl-l-valine were found to exhibit antifungal and antibacterial activities [21]. They also inhibited the apoptosis-regulating enzymes, caspase-1 and caspase-3, in a cell-free system [20], and such biological activity has prompted us to explore the inhibitory activity of the sesquiterpene lactones (**1**–**7**) toward the caspase-3 enzyme. The cytotoxic activity of **1**–**7** was evaluated via a cell viability assay using a HepG2 cancer cell line. Compounds **2**, **3** and **7** exhibited cytotoxic activity with IC_50_ values of 193.3, 50.6 and 57.0 µM, respectively, while **1**, **4**, **5** and **6** did not show activity at 200 μM. The inhibitory activity of compounds **1**–**7** toward caspase-3 was also analyzed in a cell-free system. A crude extract of caspase-3 enzyme was obtained from a cell lysate that was prepared from apoptotic HepG2 cells treated with doxorubicin (10 µM). Normally, it is known that doxorubicin can activate caspase-3 enzyme in the cells. In the present work, the presence of active caspase-3 induced by doxorubicin was confirmed by immunoblot analysis. A known caspase-3 inhibitor, Z-DEVD-fmk, was used as a positive control. As shown in Figure 3, compounds **1**–**7** did not inhibit caspase-3 activity, while Z-DEVD-fmk markedly decreased the caspase-3 activity. It should be noted that berkedrimane B (**2**) was previously found to inhibit caspase-3 with the IC_50_ value of 50 μM [20]; however, in the present study, it is inactive toward caspase-3.

## 3. Experimental Section

### 3.1. General Experimental Procedures

Melting points were measured using Buchi 535 Melting Point Apparatus, and reported without correction. IR spectra were obtained from a universal attenuated total reflectance (UATR) attachment on a Perkin-Elmer Spectrum One spectrometer, while UV-Vis spectra were obtained from Shimadzu UV-1700 PharmaSpec Spectrophotometer. Optical rotations were measured using a JASCO P-1020 polarimeter. ^1^H and ^13^C NMR spectra were recorded on a Bruker Avance 600 (operating at 600 MHz for ^1^H and 150 MHz for ^13^C). ESITOF MS spectra were obtained from a Bruker MicroTOF_lc_ spectrometer.

### 3.2. Fungal Material and Identification of the Fungus PILE 14-5

*T. minioluteus* PILE 14-5 was isolated from an unidentified marine sponge, collected in February 2009 from Pilae Bay, Phi Phi Island, Krabi Province, Thailand. The PILE 14-5 fungus was identified based on morphological characteristics and the analysis of DNA sequences of the ITS1-5.8S-ITS2 ribosomal RNA gene region. 

#### 3.2.1. Morphological Characterization

The PILE 14-5 fungus was inoculated in three-point fashion on Czapek yeast extract agar (CYA) using 90 mm plastic Petri dishes. Plates were incubated at 25, 30 and 37 °C, in darkness. Additionally, the fungus was similarly cultured on malt extract agar (MEA) at 25 and 30 °C. After 7 days of incubation, colony morphology was examined. Slide cultures on MEA were prepared for microscopic examination of sporulated fungus [14]. After 7 days incubation on CYA the PILE 14-5 fungus grew to a colony diameter of 17–18 mm at 25 °C, 20–22 mm at 30 °C and there was no growth at 37 °C. Colonies were raised at the center, sporulating area appeared greyish blue-green, with radiate groves; colony periphery were low, plane, containing white mycelia, with entire (smooth) margins, and slight production of brownish-red soluble pigment. The reverse side of the colonies was reddish brown at the center fading into brownish orange towards the dull yellow margin. Microscopically, the conidiophores were biverticillate, and the stipe wall was smooth. Conidia were smooth, ellipsoidal, 3 × 2.5 µm. Ascomata were absent. The morphological character which showed biverticillate penicilli conidial bearing structure suggested that this fungus belongs to the genus *Talaromyces*. Comparing with *Talaromyces* in the group of soluble red pigment producers, the growth rate on CYA and the inability to grow at 37 °C distinguished the PILE 14-5 from other members but were similar to those of *Talaromyces minioluteus* [14]. 

#### 3.2.2. DNA Sequence-Based Identification

The PILE 14-5 fungus was cultivated in malt extract broth for 5 days, and the mycelium was collected and washed with sterile water. Cellular DNA was extracted from the washed fungal mycelium using the FTA^®^ Plant Kit (Whatman^®^, Florham Park, New Jersey, USA) according to the manufacturer’s instructions. The ITS1-5.8S-ITS2 of the ribosomal RNA gene region was amplified from the fungal genomic DNA by PCR (GoTaq® Colorless Master Mix, Promega, Madison, WI, USA) using the ITS5 (GGAAGTAAAAGTCGTAACAAGG) and ITS4 (TCCTCCGCTTATTGATATGC) primers [25]. The thermal cycle program was as follows: 5 min at 95 °C followed by 30 cycles of 50 s at 95 °C, 40 s at 45 °C and 60 s at 72 °C, with a final extension period of 10 min at 72 °C (GeneAmp^®^ PCR System 9700, Applied Biosystems, Foster City, CA, USA). The amplified DNA fragment was purified and subjected to DNA sequencing on both strands using primers ITS5 and ITS4. The DNA sequence of the complete ITS1-5.8S-ITS2 (508 nucleotides) was submitted to the online BLASTN 2.2.30+ [26] to search for similar sequences in GenBank. The DNA sequences from type strains were selected for alignment using the ClustalW multiple sequence alignment program in the CLC Main Workbench software package version 6.6.2 (CLC bio, Aarhus, Denmark) with manual final adjustment. Phylogenetic relationship was estimated using the neighbor-joining method. Bootstrap analysis was performed with 1000 replications to determine the support for each clade.

BLAST search of the ITS1-5.8S-ITS2 DNA sequence revealed that the PILE 14-5 was related to fungi in the genus *Talaromyces*. Highly similar ITS1-5.8S-ITS2 DNA sequences from 12 reference fungal strains having 5.8S identical to that of the PILE 14-5 were retrieved for constructing a phylogenetic tree. The PILE 14-5 fungus formed a cluster with *Talaromyces minioluteus* CBS 642.68, a type strain fungus, with 96.85% ITS1-5.8S-ITS2 sequence identity. The next closely related fungus was *T. udagawae* CBS 579.72 with 94.69% sequence identity. Ten other related fungi had 84%–86% sequence identity with the PILE 14-5. Based on morphological characteristics and phylogenetic analysis with reference to current fungal taxonomy [14], this fungus was identified as *Talaromyces minioluteus* PILE 14-5 in its anamorphic form. DNA sequence data of ITS1-5.8S-ITS2 rRNA gene region of the PILE 14-5 fungus has been deposited in GenBank with an accession number of KF471124. A culture of the PILE 14-5 has been deposited at Chulabhorn Research Institute.

### 3.3. Fungal Cultivation, Extraction and Isolation of Metabolites

The fungus *T. minioluteus* was cultivated in potato dextrose broth (PDB), which was prepared in seawater instead of distilled H_2_O. The fungus was grown in a 1 L Erlenmeyer flask, each containing 250 mL of PDB; the total volume of culture broth was 5 L. Fungal culture was incubated at room temperature for 30 days (under static conditions), and culture broth was separated from cells by filtration. A broth was extracted three times, each with an equal volume of EtOAc, yielding a crude broth extract (1.8 g). A broth extract was subjected to Sephadex LH-20 column chromatography (CC) (4 × 45 cm, eluted with MeOH), to yield 20 fractions (A1–A20). Fractions from Sephadex LH-20 CC were combined by comparison of the ^1^H NMR profile of an individual fraction. Fractions A4 (428.0 mg) and A5 (391.3 mg) were combined, and this material was purified by Sephadex LH-20 CC (4 × 45 cm, eluted with MeOH), to give 10 fractions (B1–B10). Fraction B5 (523.6 mg) was further purified by Sephadex LH-20 CC (2 × 125 cm, eluted with MeOH), to yield 5 fractions (C1–C5). Fraction C1 (156.6 mg) was separated by C_18_ reversed-phase HPLC, eluted with a mixture of MeOH:H_2_O (6:4, v/v) with a flow rate of 8.5 mL/min, yielding 2.2 mg of **6** (*t*_R_ 18.4 min), 5.7 mg of **7** (*t*_R_ 20.8 min), 9.1 mg of **4** (*t*_R_ 26.0 min), 13.5 mg of **2** (*t*_R_ 29.0 min), 1.8 mg of **3** (*t*_R_ 33.0 min), 3.3 mg of **5** (*t*_R_ 35.7 min), and 45.7 mg of purpuride (**1**) (*t*_R_ 38.1 min). Fraction C2 (346.1 mg) was further purified by Sephadex LH-20 CC (2 × 125 cm), eluted with MeOH, to yield 3 fractions (D1–D3). Fraction D2 (181.9 mg) was further separated by C_18_ reversed-phase HPLC, using a mixture of MeOH:H_2_O (6:4, v/v) as an eluent (a flow rate of 8.5 mL/min), giving 5.6 mg of **7** (*t*_R_ 20.8 min), 15.8 mg of **4** (*t*_R_ 26.0 min), 35.6 mg of berkedrimane B (**2**) (*t*_R_ 29.0 min), 2.9 mg of **3** (*t*_R_ 33.0 min), 2.4 mg of **5** (*t*_R_ 35.7 min), and 48.6 mg of purpuride (**1**) (*t*_R_ 38.1 min). The ^1^H and ^13^C NMR spectra for new metabolites **3**–**7** can be found as supplemental material.

### 3.4. Spectroscopic Data of Compounds

Purpuride (**1**): [α]^28^_D_ +77.1 (*c* 0.86, CHCl_3_), Lit. [19,21] +79.3 (*c* 0.1, CHCl_3_).

Berkedrimane B (**2**): [α]^28^_D_ −42.5 (*c* 0.42, CHCl_3_), Lit. [20] −15.3 (*c* 0.0145, CHCl_3_).

Minioluteumide A (**3**): Needle-like crystals; m. p. 176.9−177.0 °C; [α]^28^_D_ −12.0 (*c* 0.36, CHCl_3_); UV (MeOH) λ_max_ (log ε) 214 (3.4); IR ν_max_ 3335, 2925, 1733, 1661, 1536, 1462, 1374, 1256, 1158, 1092, 1027, 1006, 734 cm^−1^; ^1^H and ^13^C NMR data, see Table 1; ESITOF MS: *m*/*z* 426.2063 [M + H]^+^ (calcd for C_22_H_33_ClNO_5_, 426.2047).

Minioluteumide B (**4**): Off-white amorphous solid; [α]^28^_D_ +19.3 (*c* 0.44, CHCl_3_); UV (MeOH) λ_max_ (log ε) 211 (3.5); IR ν_max_ 3336, 2961, 2933, 1733, 1661, 1532, 1461, 1373, 1254, 1142, 1092, 1039, 1004, 893, 735 cm^−1^; ^1^H and ^13^C NMR data, see Table 1; ESITOF MS: *m*/*z* 408.2401 [M + H]^+^ (calcd for C_22_H_34_NO_6_, 408.2386).

Purpuride B (**5**): Off-white amorphous solid; [α]^28^_D_ +20.3 (*c* 0.32, CHCl_3_), Lit. [21] +3.8 (*c* 0.07, CHCl_3_).

Minioluteumide D (**6**): Off-white amorphous solid; [α]^28^_D_ +14.5 (*c* 0.14, CHCl_3_); UV (MeOH) λ_max_ (log ε) 202 (3.4), 213(3.3); IR ν_max_ 336, 2924, 2854, 1734, 1661, 1541, 1463, 1373, 1240, 1160, 1032, 1007, 803 cm^−1^; ^1^H and ^13^C NMR data, see Table 2; ESITOF MS: *m*/*z* 408.2394 [M + H]^+^ (calcd for C_22_H_34_NO_6_, 408.2386).

Minioluteumide E (**7**): Off-white amorphous solid; [α]^28^_D_ +28.1 (*c* 0.45, CHCl_3_); UV (MeOH) λ_max_ (log ε) 203 (3.5); IR ν_max_ 3356, 2960, 2930, 1745, 1660, 1538, 1459, 1374, 1264, 1147, 1093, 1072, 1027, 968 cm^−1^; ^1^H and ^13^C NMR data, see Table 2; ESITOF MS: *m*/*z* 408.2399 [M + H]^+^ (calcd for C_22_H_34_NO_6_, 408.2386).

### 3.5. Single Crystal X-Ray Analysis

Single crystal X-ray diffraction data were collected at 296(2) K on a Bruker X8 PROSPECTOR KAPPA CCD diffractometer using an IμS X-ray microfocus source with multilayer mirrors, yielding intense monochromatic Cu-Kα radiation (λ = 1.54178 Å). The structure was solved using SHELXS-97 [27] and refined using full-matrix least squares on *F*^2^ with SHELXL-97 [27]. A water site with occupancy factor 0.25 is a trace impurity in the crystallization solvents; its H-atoms could not be located.

Crystal Data of **3**: C_22_H_32_ClNO_5_·0.25H_2_O, MW = 430.44, light yellow, long needle-like crystal: 0.08 × 0.08 × 0.45 mm^3^, orthorhombic space group *P*2_1_2_1_2_1_, *a* = 9.9172(3) Å, *b* = 14.7327(4) Å, *c* = 15.4578(4) Å, *V* = 2258.49(11) Å^3^, *Z* = 4, *D_x_* = 1.264 g cm^−3^, μ(Cu-Kα) = 1.772 mm^−1^, *F*(000) = 920. Unique reflections: 3180 (*R*_int_ = 0.0213). The final *R*_1_(*F*^2^) = 0.0396 and *wR*(*F*^2^) = 0.1129 for 2838 reflections with *F*^2^ > 2σ(*F*^2^). Flack parameter [22]: 0.05(2). CCDC-1041970 contains the supplementary crystallographic data for this paper. These data can be obtained free of charge from the Cambridge Crystallographic Data Centre [28].

### 3.6. Bioassays

#### 3.6.1. Cell Culture

Human hepatocellular carcinoma cell line, HepG2, was obtained from the American Type Culture Collection (ATCC, Rockville, MD, USA). HepG2 cell line was cultured in DMEM supplemented with 10% fetal bovine serum and antibiotics, and maintained at 37 °C in a humidified atmosphere of 5% CO_2_.

#### 3.6.2. Cell Viability Assay

Viability of cells after treatment with compounds was determined by MTT assay as previously described by Chiablaem *et al.* [29]. HepG2 cells were seeded at 1 × 10^4^ cells/100 μL/well in 96-well plates, and incubated for 24 h. HepG2 cells were treated with media containing test compounds (100 μL) and further incubated for 72 h. The media in each well were then replaced with media (100 μL) containing MTT (0.5 mg/mL) and incubated for 2 h. Subsequently, the media containing MTT were removed, and DMSO was added (100 μL/well). An absorbance was measured at 550 nm and subtracted with an absorbance at 650 nm, using a microplate reader. Experiments were performed in triplicate wells. Data are expressed as percent viability compared with a control. Doxorubicin (Doxo) and galactosamine (GalN) were used as reference compounds, showing the cytotoxic activity with respective IC_50_ values of 0.52 µM and 13.17 mM. 

#### 3.6.3. Caspase-3 Activity Assay

Caspase-Glo 3/7 Assay kit (Promega, Madison, WI, USA) was used to measure caspase-3 activity, and the assay was performed according to the manufacturer’s instructions. Lysate of HepG2 cells treated with a standard drug, doxorubicin, was used as a source of caspase-3 enzyme. To prepare a crude caspase-3 enzyme, HepG2 cells were seeded at 6 × 10^5^ cells in 6 well plates (2 mL/well), and incubated for 24 h at 37 °C in a CO_2_ incubator. Subsequently, caspase-3 enzyme was activated by treating the cells with 10 µM of doxorubicin for 48 h. After that, media were removed and the cells were washed twice with phosphate buffer saline and lysed in 500 μL/well passive lysis buffer (PLB, Promega) containing protease inhibitor cocktail (Sigma, St. Louis, MO, USA). The plates were gently agitated on ice for 20 min, then cell lysate was collected and centrifuged at 10,000× *g*, at 4 °C for 5 min, supernatant was collected and kept at −80 °C until use. To detect caspase-3 activity, 100 μL of a reaction mixture composing of 50 μL of PLB containing the cell lysate (5 μg protein/mL) and test compounds, and 50 μL of Caspase-Glo 3/7 reagent, was incubated at room temperature for 1 h. The peptide (5 µM), Z-DEVD-fmk, a known caspase-3 inhibitor, was used as a positive control. Luminescence was measured by using a luminometer (ATTO, Tokyo, Japan). Data are expressed as percentage of caspase-3 activity compared with a control.

## 4. Conclusions

Chemical investigation of a broth extract of the marine fungus *T. minioluteus* led to the isolation of four new sesquiterpene lactones **3**, **4**, **6** and **7**, together with three known compounds, purpuride (**1**), berkedrimane B (**2**) and purpuride B (**5**). The isolated compounds were sesquiterpene lactones conjugated with *N*-acetyl-l-valine, which are rarely found in nature. Among the isolated fungal metabolites, compounds **2**, **3** and **7** showed cytotoxic activity with IC_50_ ranges of 50.6–193.3 µM, but all compounds (**1**–**7**) did not inhibit caspase-3 activity. New bioactive secondary metabolites with diverse chemical skeletons have recently been isolated from marine fungi [30,31,32]. The present work underscores that marine fungi are important sources of bioactive natural products. 

## Supplementary Files

Supplementary File 1
