# Supplementary material for: Drimane Sesquiterpene-Conjugated Amino Acids from a Marine Isolate of the Fungus Talaromyces minioluteus (Penicillium Minioluteum)
Source: Mar Drugs. 2015 Jun 5;13(6):3567–80. doi: 10.3390/md13063567 (PMC4483645; doi:10.3390/md13063567)
Supplement: Supplementary File 1 [file marinedrugs-13-03567-s001.pdf]

Supplementary Information

^{1}H and ^{13}C spectra of compounds **3**–**7**.

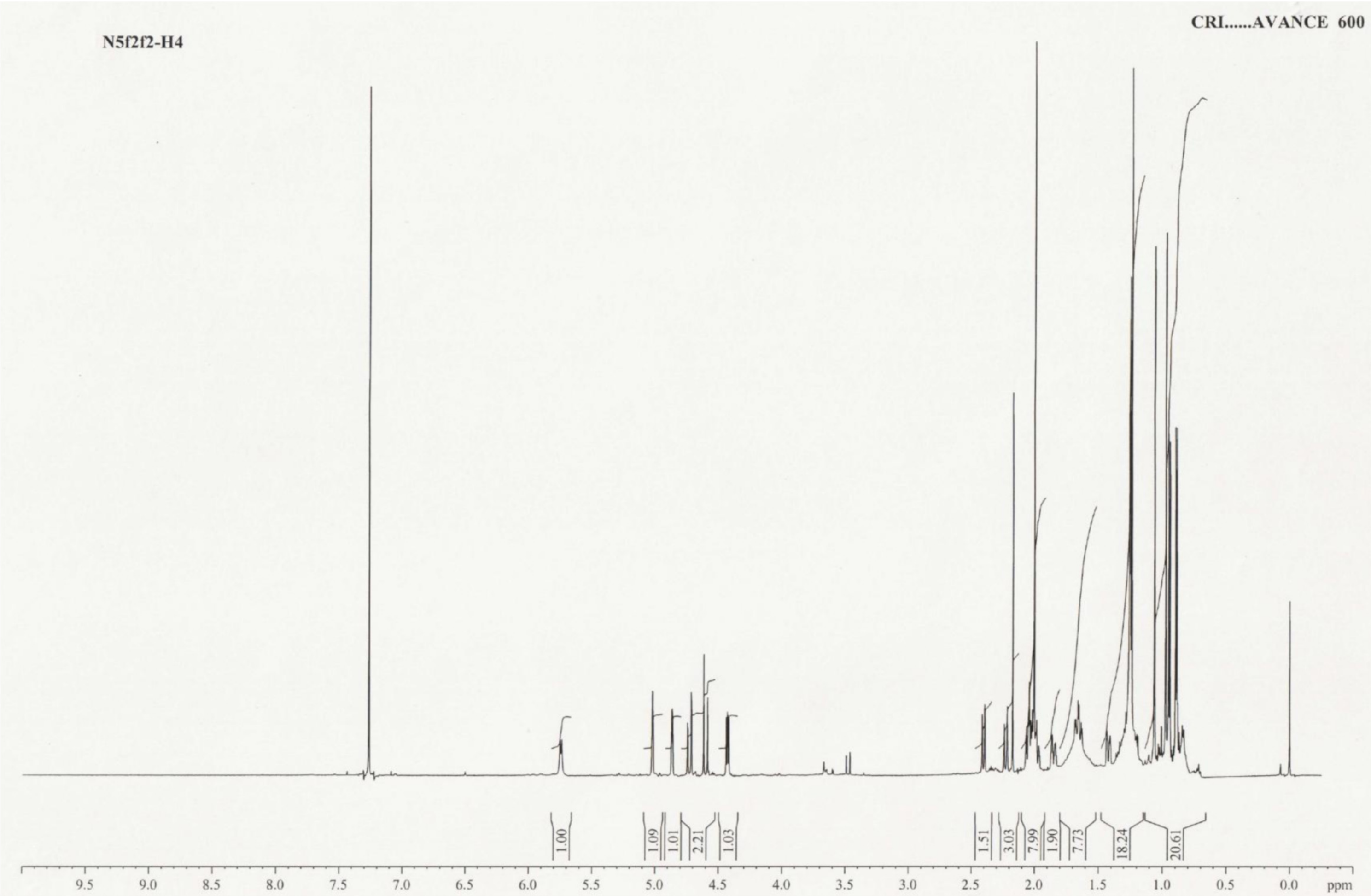

Figure S1. ^{1}H NMR spectrum ($CDCl_3$) of compound **3**.

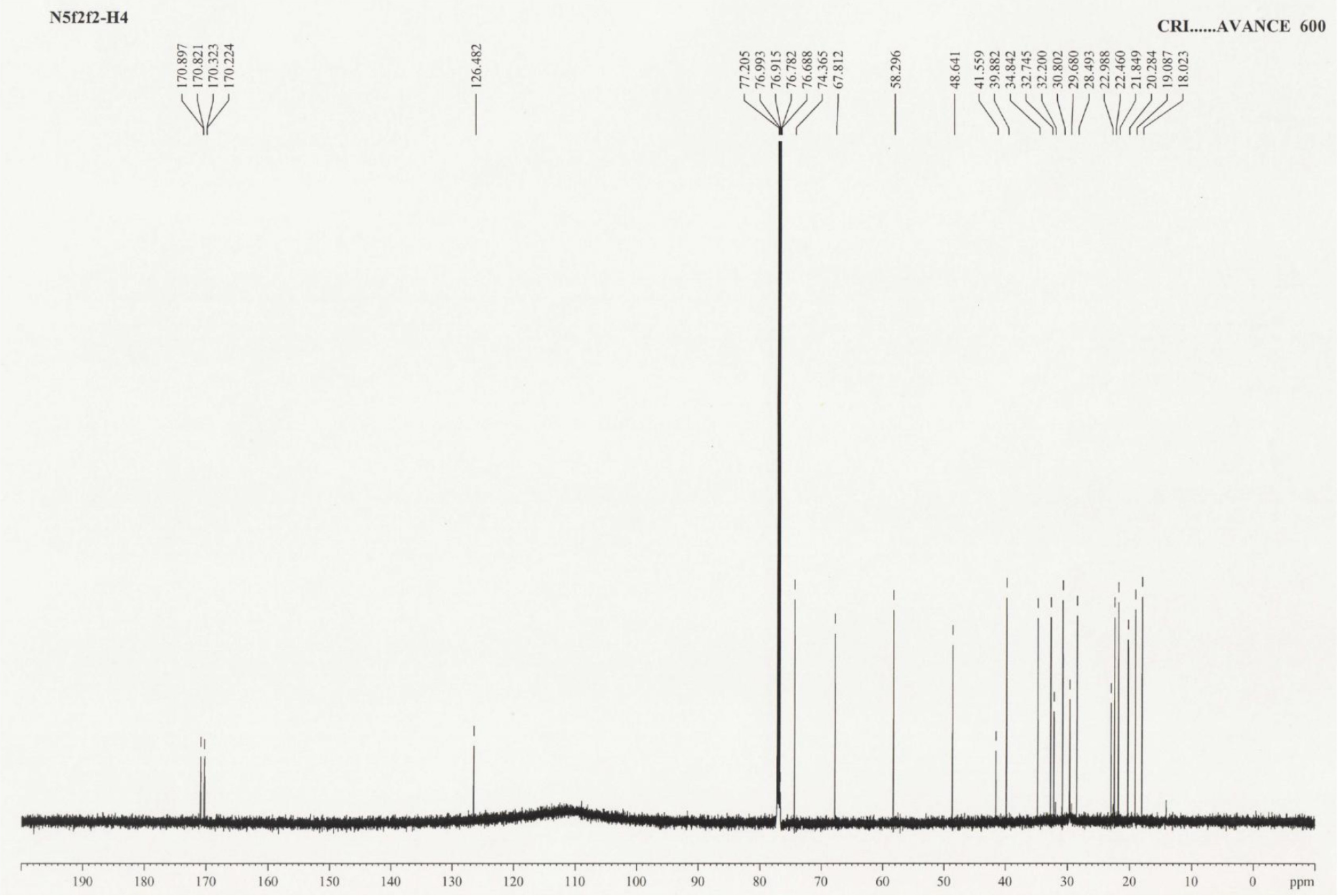

Figure S2. ^{13}C NMR spectrum ($CDCl_3$) of compound **3**.

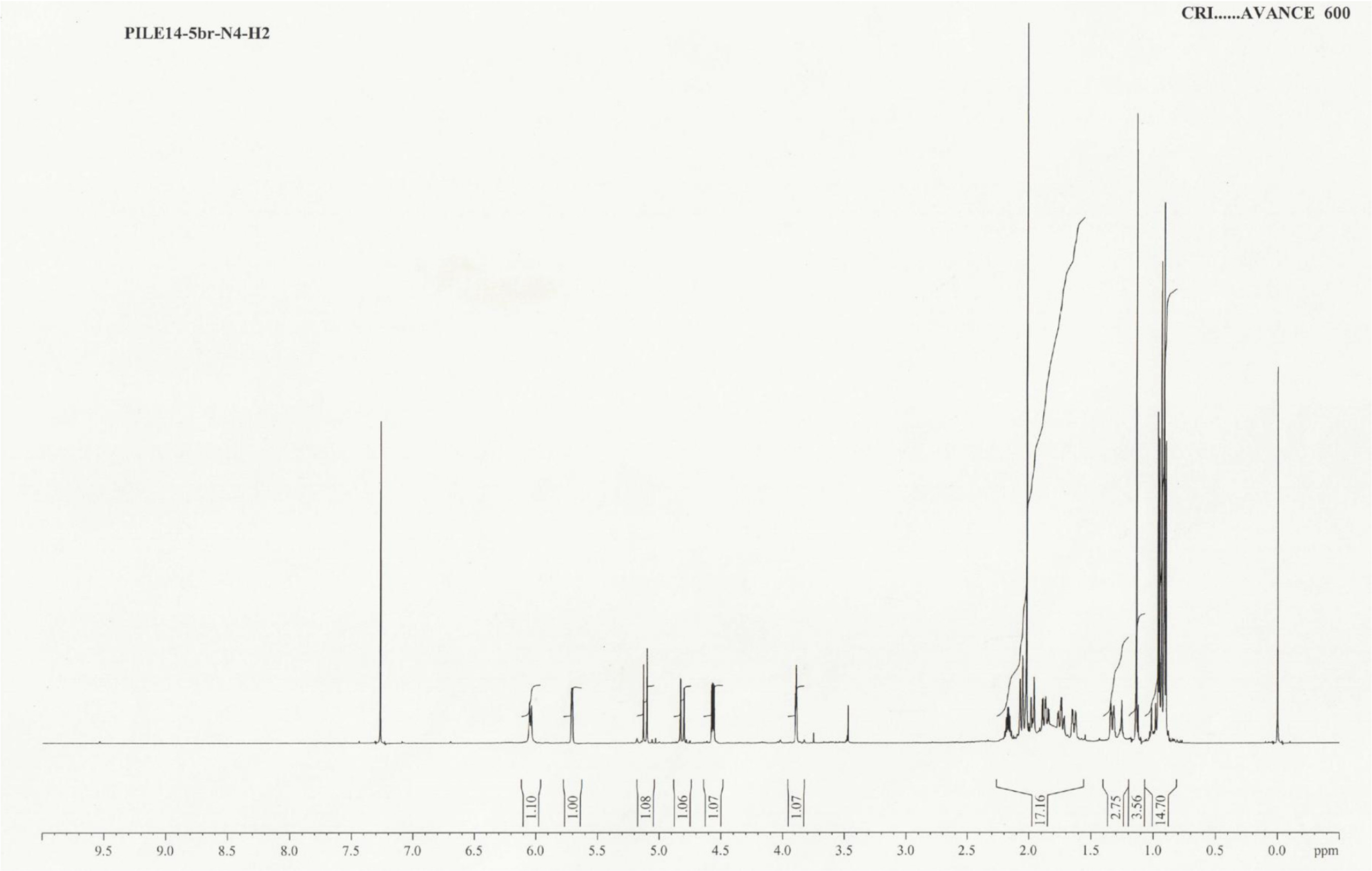

Figure S3. ^{1}H NMR spectrum ($CDCl_3$) of compound **4**.

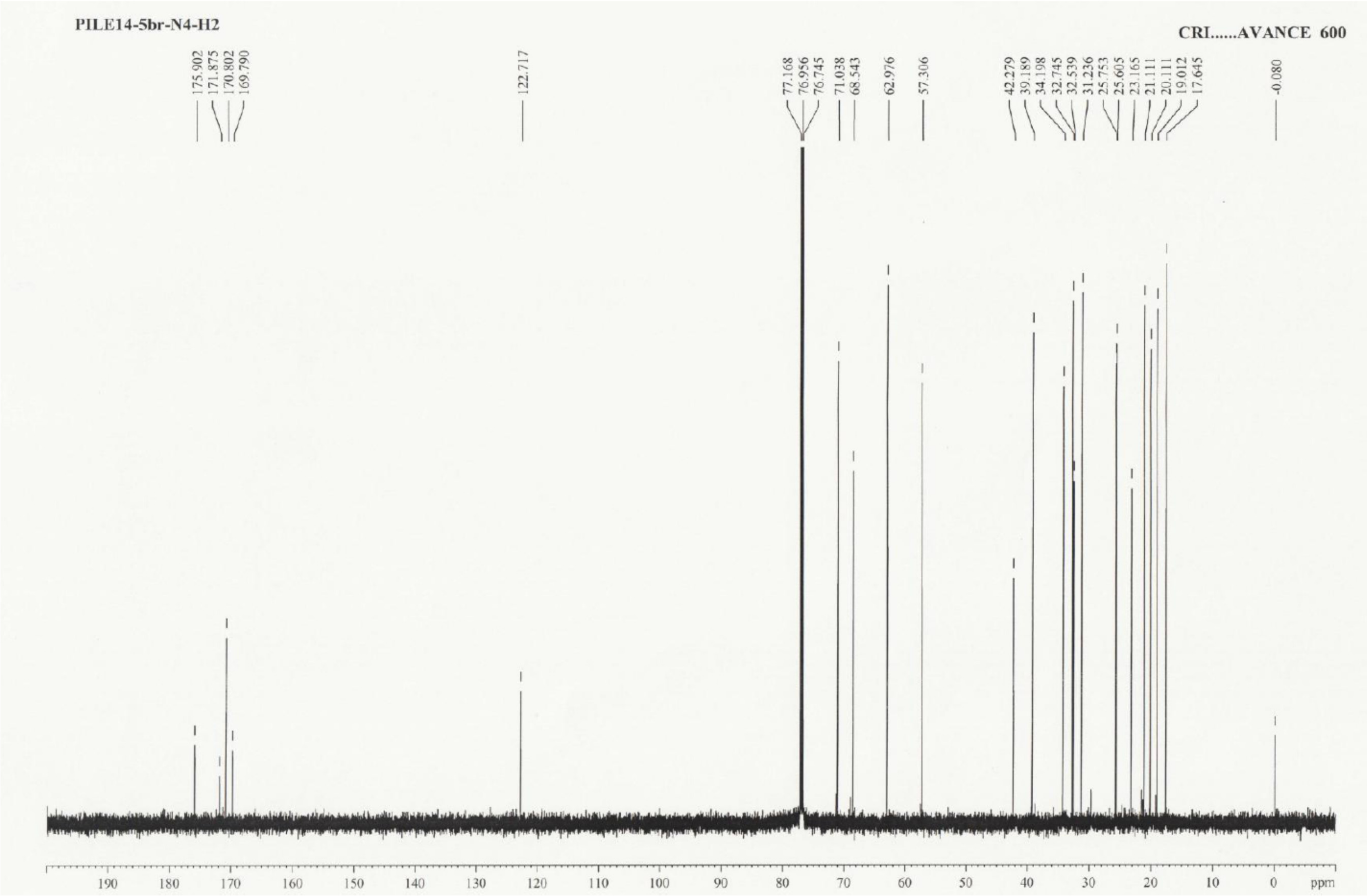

Figure S4. ^{13}C NMR spectrum ($CDCl_3$) of compound **4**.

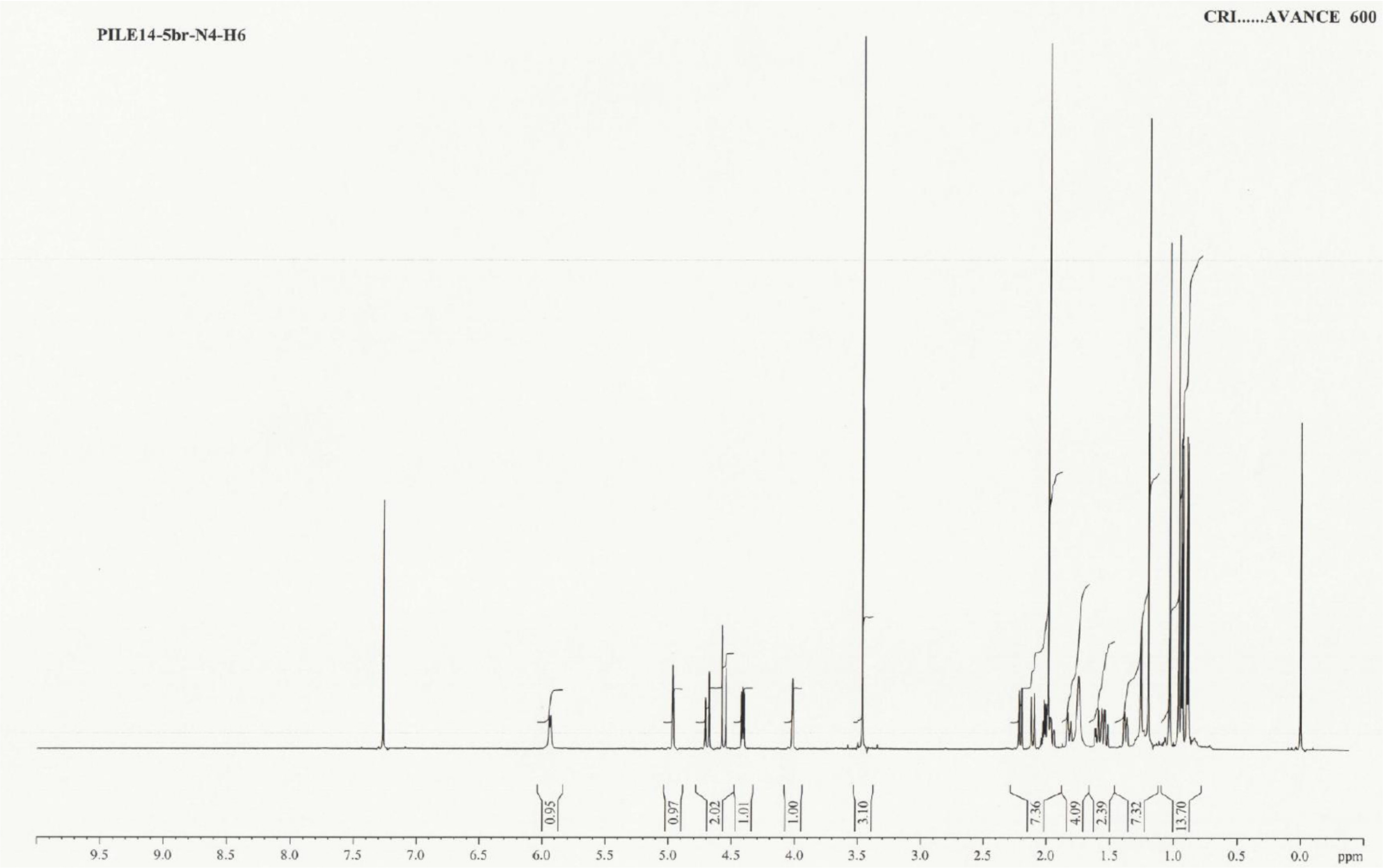

Figure S5. ^{1}H NMR spectrum ($CDCl_3$) of compound **5**.

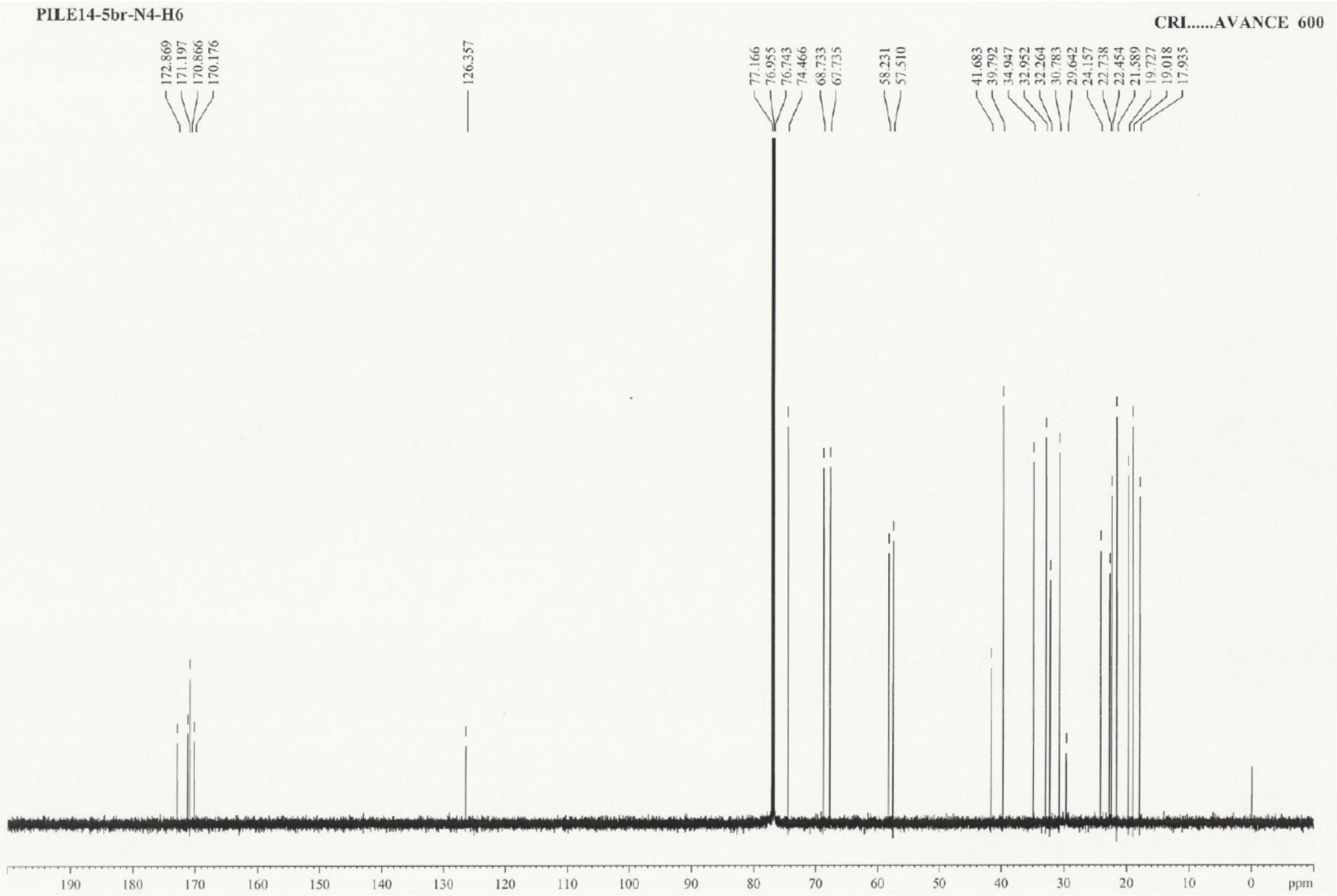

Figure S6. ^{13}C NMR spectrum ($CDCl_3$) of compound **5**.

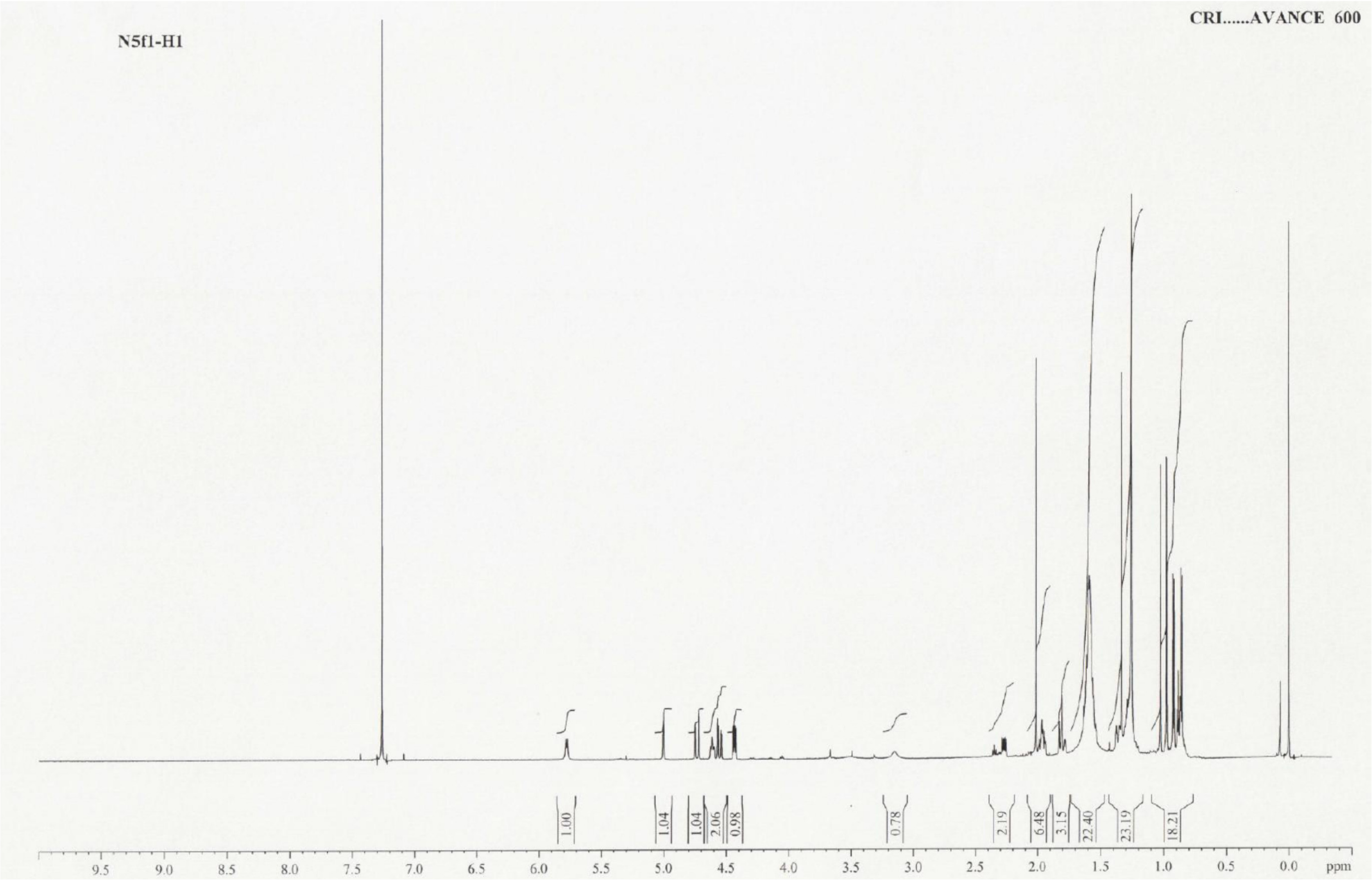

Figure S7. ^{1}H NMR spectrum ($CDCl_3$) of compound **6**.

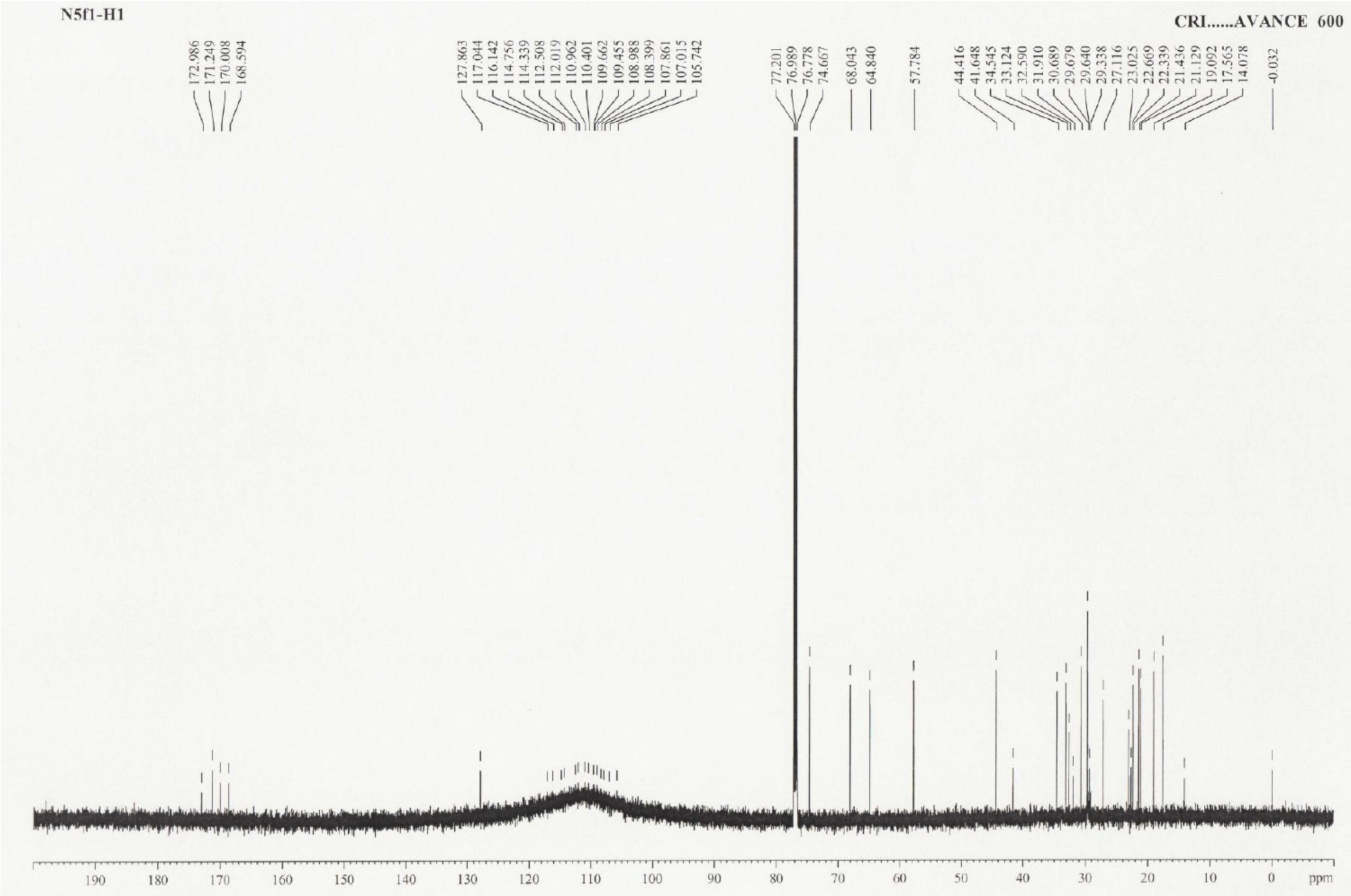

Figure S8. ^{13}C NMR spectrum ($CDCl_3$) of compound **6**.

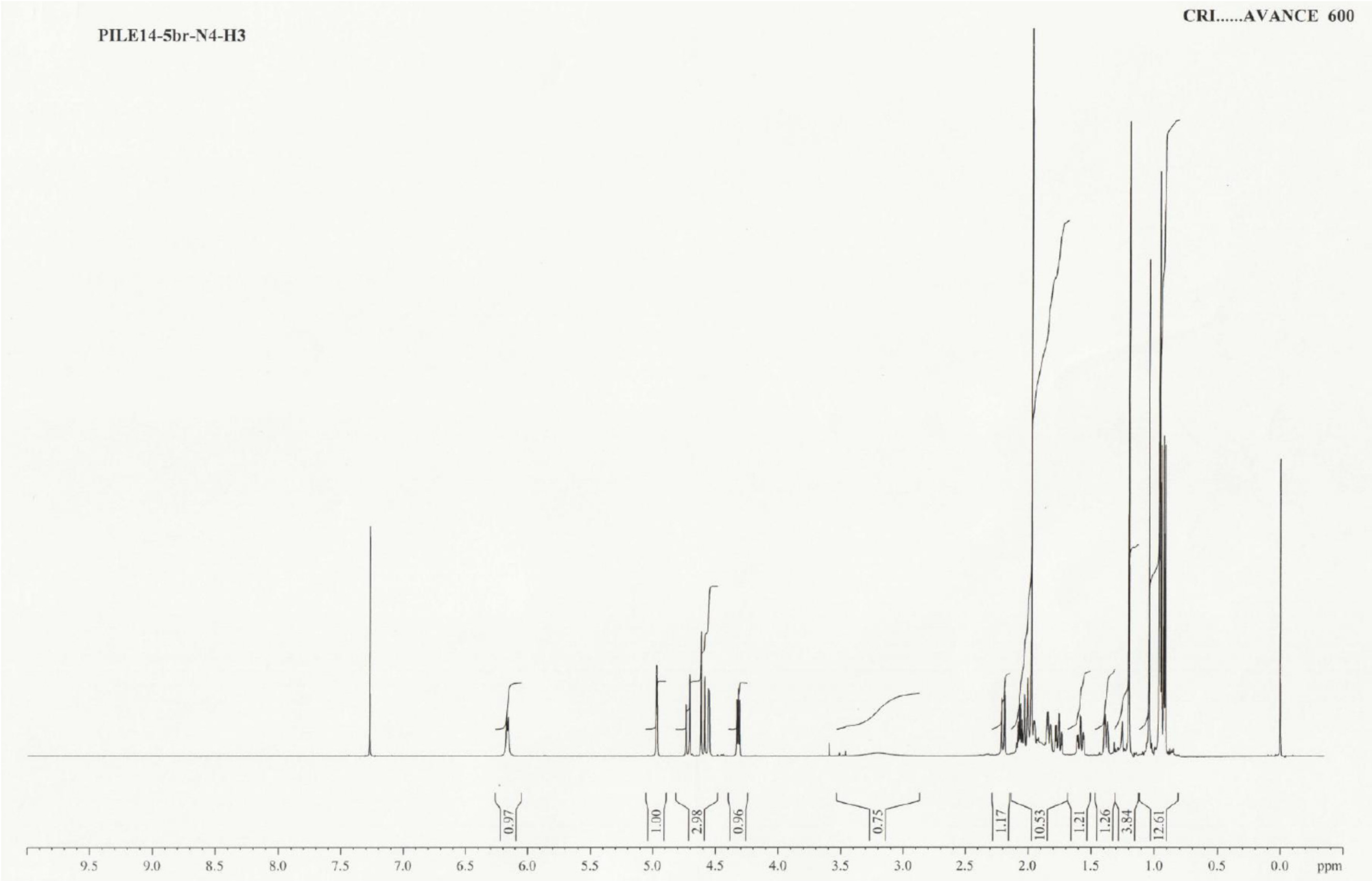

Figure S9. ^{1}H NMR spectrum ($CDCl_3$) of compound **7**.

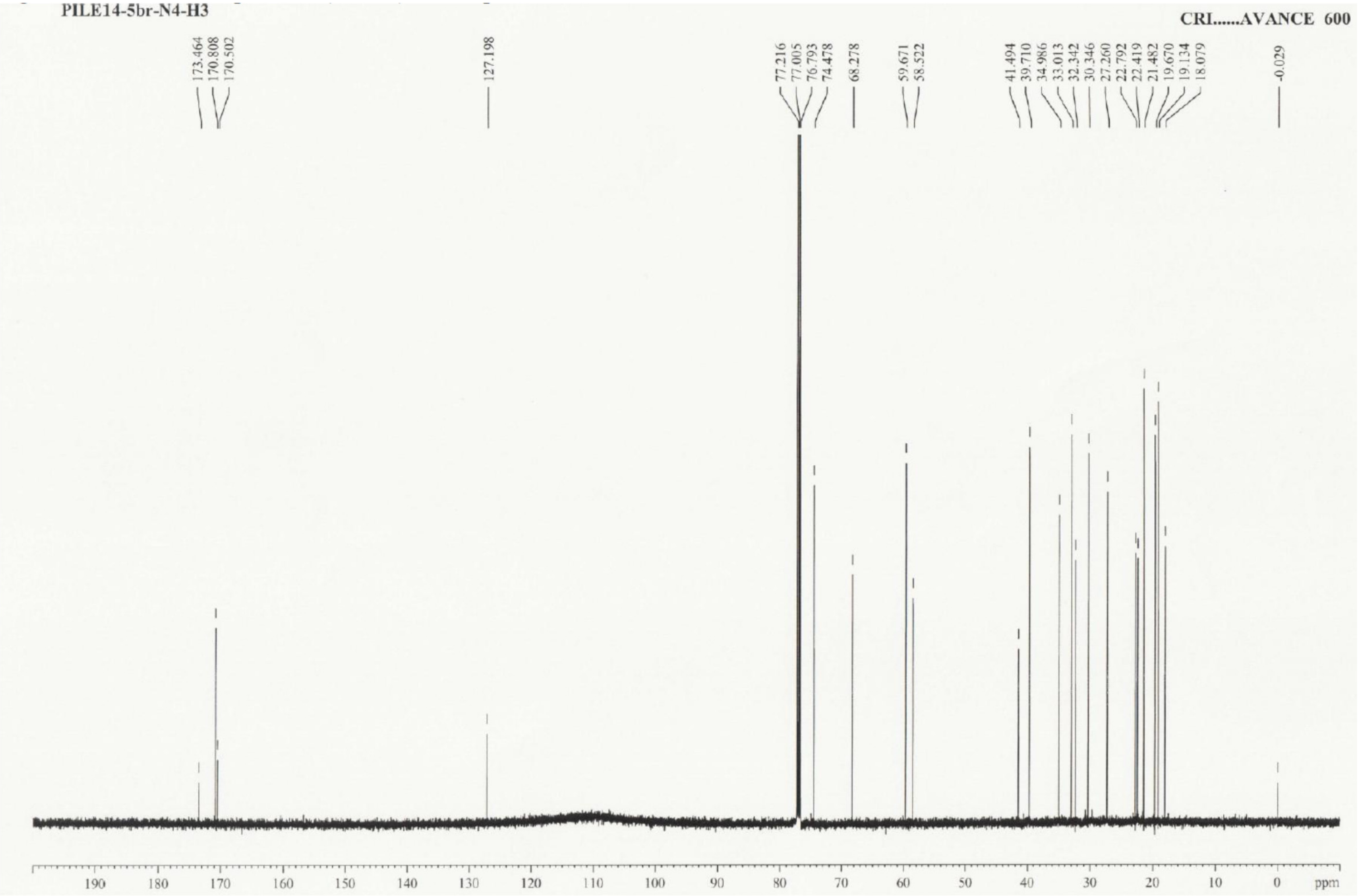

Figure S10. ^{13}C NMR spectrum ($CDCl_3$) of compound **7**.

© 2015 by the authors; licensee MDPI, Basel, Switzerland. This article is an open access article distributed under the terms and conditions of the Creative Commons Attribution license (http://creativecommons.org/licenses/by/4.0/).
